# A review on antitumor effect of pachymic acid

**DOI:** 10.1097/MD.0000000000039752

**Published:** 2024-09-20

**Authors:** Yubo Xiao, Zhaotun Hu, Hang Liu, Xinglin Jiang, Taimei Zhou, Haiying Wang, Heng Long, Ming Li

**Affiliations:** a School of Public Health and Laboratory Medicine, Hunan University of Medicine, Huaihua, China; b Key Laboratory of Research and Utilization of Ethnomedicinal Plant Resources of Hunan Province College of Biological and Food Engineering, Huaihua University, Huaihua, China; c Department of Breast and Thyroid Surgery, First People’s Hospital of Huaihua City, Huaihua, China; d Department of Histology and Embryology, Hunan University of Medicine, Huaihua, China.

**Keywords:** anti-tumor, apoptosis, cell cycle, combined therapy, pachymic acid, Poria cocos

## Abstract

Poria cocos, also known as Jade Ling and Songbai taro, is a dry fungus core for Wolfiporia cocos, which is parasitic on the roots of pine trees. The ancients called it “medicine of four seasons” because of its extensive effect and ability to be combined with many medicines. Pachymic acid (PA) is one of the main biological compounds of Poria cocos. Research has shown that PA has various pharmacological properties, including anti-inflammatory and antioxidant. PA has recently attracted much attention due to its anticancer properties. Researchers have found that PA showed anticancer activity by regulating apoptosis and the cell cycle in vitro and in vivo. Using PA with anticancer drugs, radiotherapy, and biomaterials could also improve the sensitivity of cancer cells and delay the progression of cancer. The purpose of this review was to summarize the anticancer mechanism of PA by referencing the published documents. A review of the collected data indicated that PA had the potential to be developed into an effective anticancer agent.

**Key Points:**

1. Pachymic acid has an anticancer effect by inducing apoptosis of various cancer cells.
2. Pachymic acid plays an anticancer role by inducing cell cycle arrest of various cancer cells.
3. Pachymic acid could reduce the drug resistance of cancer cells by combining with other anticancer drugs.

## 1. Introduction

Cancer is becoming one of the leading causes of death in both developed and developing countries. Currently, conventional drugs, surgery, radiotherapy, and chemotherapy are the standard treatments.^[1]^ Traditional treatment methods are problematic due to their toxicity and resistance to cancer cells. Cancer cells’ metastasis and recurrence rates are very high, even after the standard treatment plan has been completed. People are constantly trying to find innovative therapy to treat a variety of cancers. An emerging method for treating tumors is genetic therapy, which involves modifying and suppressing cancer genes.^[2]^ The use of gene therapy for medical purposes is still fraught with security problems, and it has long been a controversial issue in ethics to prohibit human trials. A Nobel Prize was awarded to researchers who proposed the theory of using the immune system to treat cancer in 2018.^[3]^ Multiple immunotherapies are not, however, considered satisfactory for the long-term treatment of solid tumors. Various immune cells and immunosuppressive cytokines regulate immunosuppression in tumor microenvironments, and tumor-specific immune cells are poorly targeted and penetrate the tumor microenvironment. Although oncolytic viruses have been incorporated into this therapy to improve the penetration of targeted immune cells, it is still in clinical trials.^[4]^

Traditional Chinese medicine (TCM) has been practiced for thousands of years and is now widely recognized as a viable alternative cancer treatment.^[5]^ It is known that compounds derived from plants have several advantages, including the ability to target multiple targets, being low in cost, relatively nontoxic, and being readily available.^[6]^ Zhao et al^[7]^ were increasingly examining herbal medicine prescriptions and monomers. Icariin, for example, is known to have anticancer activity against a variety of cancer cells by modulating apoptosis, cell cycle, antiangiogenesis, antimetastasis, and immune regulation.^[8]^ Astragalus polysaccharides, *Ganoderma lucidum* polysaccharides, and *Salvia miltiorrhiza* polysaccharides have been proven to play a therapeutic role in a variety of cancers from the perspective of immunity by regulating immune cells and promoting the proliferation of T cells and natural killer cells.^[9]^ Therefore, TCM has a great deal of potential to be used in the treatment of cancer.

Poria cocos (P. cocos) is also called Yuling or Songbai taro and is predominantly produced in Guizhou, Yunnan, Hunan, Hubei, and other provinces in China. It is a dry fungus core for Wolfiporia cocos, a fungus of the family Poromycetes. According to TCM, P. cocos promotes edema and oliguria, strengthens the spleen, and calms the heart. A wide variety of symptoms may be treated with it, including edema, urine deficiency, phlegm, dizziness, palpitations, spleen deficiency, food deficiency, loose stools, diarrhea, restlessness, palpitations, and insomnia.^[10]^ There is a TCM saying “Nine P. cocos in Ten Directions.”^[11]^ Thus, P. cocos is widely used in the field of health food as a functional raw material with homology with both food and medicine. P. cocos contains polysaccharides, triterpenoids, sterols, and other chemical components. Triterpenoids found in P. cocos have been shown to be natural retinol X receptor selective agonists with potential anticancer properties.^[12]^ One of these components is pachymic acid (PA), which is the wool sterol-8-ene triterpene (C_33_H_52_O_5_). It is one of the principal triterpene components of P. cocos and has anti-inflammatory and antioxidant effects. A study found that PA inhibited inflammation and apoptosis in the lungs of rats suffering from pneumonia by activating nuclear factor kappa-B and mitogen-activated protein kinase pathways.^[13]^ According to Laban et al, PA regulated iron homeostasis, resisted lipid peroxidation, and inhibited iron death in acute renal injury.^[14]^ Additionally, PA is an effective antitumor agent. A decade ago, researchers established that PA could reduce the expression of downstream protein kinase B (AKT), weaken cancer cells’ activity, and limit cancer growth by suppressing the phospholipase A2 family of arachidonic acid-generating enzymes.^[15,16]^ Hong et al^[17]^ demonstrated that PA inhibited PITPNM3 phosphorylation to reduce p-PITPNM3 and chemokine (C-C motif) receptor 8 binding, which inhibited BC spread. PA effectively inhibited BC cells’ invasiveness through downregulation of matrix metalloproteinase-9 expression mediated by PA’s suppressive effect on nuclear factor kappa-B signaling.^[18]^ These studies were published earlier and only performed cell experiments to test these conclusions. Recent studies have been conducted in vivo and in vitro regarding new mechanisms of PA’s anticancer effects. PA-treated cervical cancer mice showed inhibitory effects on tumor growth, increased levels of apoptosis, endoplasmic reticulum (ER) stress, and increased reactive oxygen species (ROS) levels in tumor tissues.^[19]^ Furthermore, PA was found to be effective in the treatment of gastric cancer (GC), pancreatic cancer, and others.^[20,21]^ The purpose of this review was to better understand the potential mechanism of anticancer activity of PA and to provide direction for future discoveries and researches on anticancer drugs.

## 2. Apoptotic activity of PA

Apoptosis is a finely programmed process of cell death in which cells silently dismantle and actively participate in several operations such as immune response, differentiation, and cell growth.^[22]^ Caspases and the B cell lymphoma-2 (Bcl-2) family are involved in several apoptosis mechanisms.^[23]^ The process of cell apoptosis is mostly initiated by caspase-3, -6, -7, and thousands of related proteins are hydrolyzed by them.^[24]^ Bcl-2 family interactions between family members determine permeabilization of the mitochondrial outer membrane and subsequent cell death.^[25]^ Apoptosis of autonomic cells is known to inhibit tumor growth, constituting a common mechanism for tumor inhibition.^[26]^

Apoptosis can be initiated by 2 main pathways: the extrinsic and the intrinsic. In the intrinsic apoptosis pathway, apoptosis-inducing Bcl-2 family members such as Bcl-2-associated X (Bax), Bcl-2 antagonist killer 1 (BAK), Bcl-2 interacting mediator of cell death (BIM), BH3-interacting domain death agonist, and p53-upregulated mediator of apoptosis (PUMA) promoted release of cytochrome c (Cyt-c) in mitochondria. Bcl-2 and B-cell lymphoma-extra large (Bcl-XL) were also negatively regulated by Bax, BAK, BIM, BH3-interacting domain death agonist, and PUMA.^[27]^ As a result of treating various types of cancer (Table 1), PA activated pro-apoptotic proteins (Cyt-c, Bax, caspase-3, -8, and -9) and inhibited antiapoptotic proteins (Bcl-2 and Bcl-XL).^[21,31,33,36]^

**Table 1:** Apoptotic activity of PA and its derivatives.

Cell line		Study type	Mechanism	Reference
Human hepatoma cells (HepG2 and Huh7 cell)		In vitro	PA triggered cell apoptosis by increasing caspase-3 and caspase-9 cleavage, upregulating Bax and Cyt-c expression, while reducing the expression of Bcl-2.	^[21]^
Human cervical cancer cells (Hela cells)		In vivo and in vitro	The ROS generation, MMP change, ATP depletion, and apoptosis were increased by PA treatment. PA triggered the expression of Cyt-c in HeLa cells. And the apoptosis-inducing factor also decreased.	^[19]^
Human gastric cancer cells	SGC-7901 and MKN-49P	In vivo and in vitro	PA treatment induces the expression of pro-apoptotic factor Bax by inhibiting hypoxia/HIF-1.	^[20]^
	SGC-7901 and MKN-49P	In vivo and in vitro	PA-induced cell apoptosis by increasing the expressions of caspase-3, PARP, and Bax and suppressing the expression of Bcl-2 and mitochondrial capacity of GC cell lines.	^[28]^
	SGC-7901 cells	In vitro	Bax, Cyt-c, and caspase-3 were markedly increased, JAK2/STAT3 was inactivated, and Bcl-2 expression was decreased following PA treatment.	^[29]^
Human pancreatic cancer cells (PANC-1 and MIA PaCa-2)		In vivo and in vitro	PA promoted the expression of XBP-1s, ATF4, CHOP, and p-eIF2α in pancreatic cancer cells through ER stress, which promoted ER stress and led to apoptosis.	^[30]^
Human bladder cancer cells (EJ cells)		In vitro	PA-induced activation of caspase-3, caspase-8, and caspase-9. PA upregulated Bax and Bad, downregulated Bcl-2 and Bcl-XL, and released cytochrome c. TNF-related apoptosis-inducing ligand, ROS, and death receptor 5 were upregulated by PA.	^[31]^
Human lung cancer cells (NCI-H23 and NCI-H460)		In vivo and in vitro	PA-induced ROS production. PA activated ER stress. The Bcl-2 was decreased, and Bax was increased. The expression of ATF4, ATF6, XBP-1, and CHOP was increased with PA treatment.	^[32]^
Human osteosarcoma cells (self-cultured cell line)		In vitro	PA-induced apoptosis in primary osteosarcoma cells increased the expression of cleaved caspase-3 and cleaved PARP.	^[33]^
Human nasopharyngeal carcinoma cells (CNE-1, CNE-2)		In vitro	PA increased the apoptosis rate of cancer cells. DNA damage-related proteins p-ATM, p-Chk-1, p-ATR, p-Chk-2, PARP, and P-histone H2AX showed an upward trend with PA treatment.	^[34]^
Breast carcinoma cells (SK-BR-3)		In vitro	PA inhibited HK2 activity, downregulating glycolysis. PA promoted the release of Cyt-C and ROS, depletion of ATP, and induced mitochondrial apoptosis.	^[35]^
Breast cancer cells (MDA-MB-231)		In vivo and in vitro	PA downregulated Bcl-2, increased the expression of Bax, and promoted the release of Cyt-c and the activation of cleaved caspase-3, caspase-9, and caspase-8.	^[36]^

ATF4 = activating transcription factor 4, ATP = adenosine triphosphate, Bax = Bcl-2-associated X, Bcl-2 = B cell lymphoma-2, Bcl-XL = B-cell lymphoma-extra large, CHOP = C/EBP homologous protein, Cyt-c = cytochrome c, ER = endoplasmic reticulum, GC = gastric cancer, HIF-1 = hypoxia-inducible factor-1, H2AX = phosphorylation of histone H2A variant H2AX, HK2 = hexokinase II, JAK2 = Janus kinase 2, MMP = mitochondrial membrane potential, PA = pachymic acid, PARP = poly (ADP-ribose) polymerase, p-ATMN = phosphorylated ataxia telangiectasia mutated proteins, p-ATR = p-serine/threonine-protein kinase ATR, p-Chk = p-serine/threonine-protein kinase Chk, p-eIF2α = p-eukaryotic initiation factor 2α, ROS = reactive oxygen species, STAT3 = signal transducer and activator of transcription 3, TNF = tumor necrosis factor, XBP-1 = α-X-box binding protein 1.

**Figure 1.**
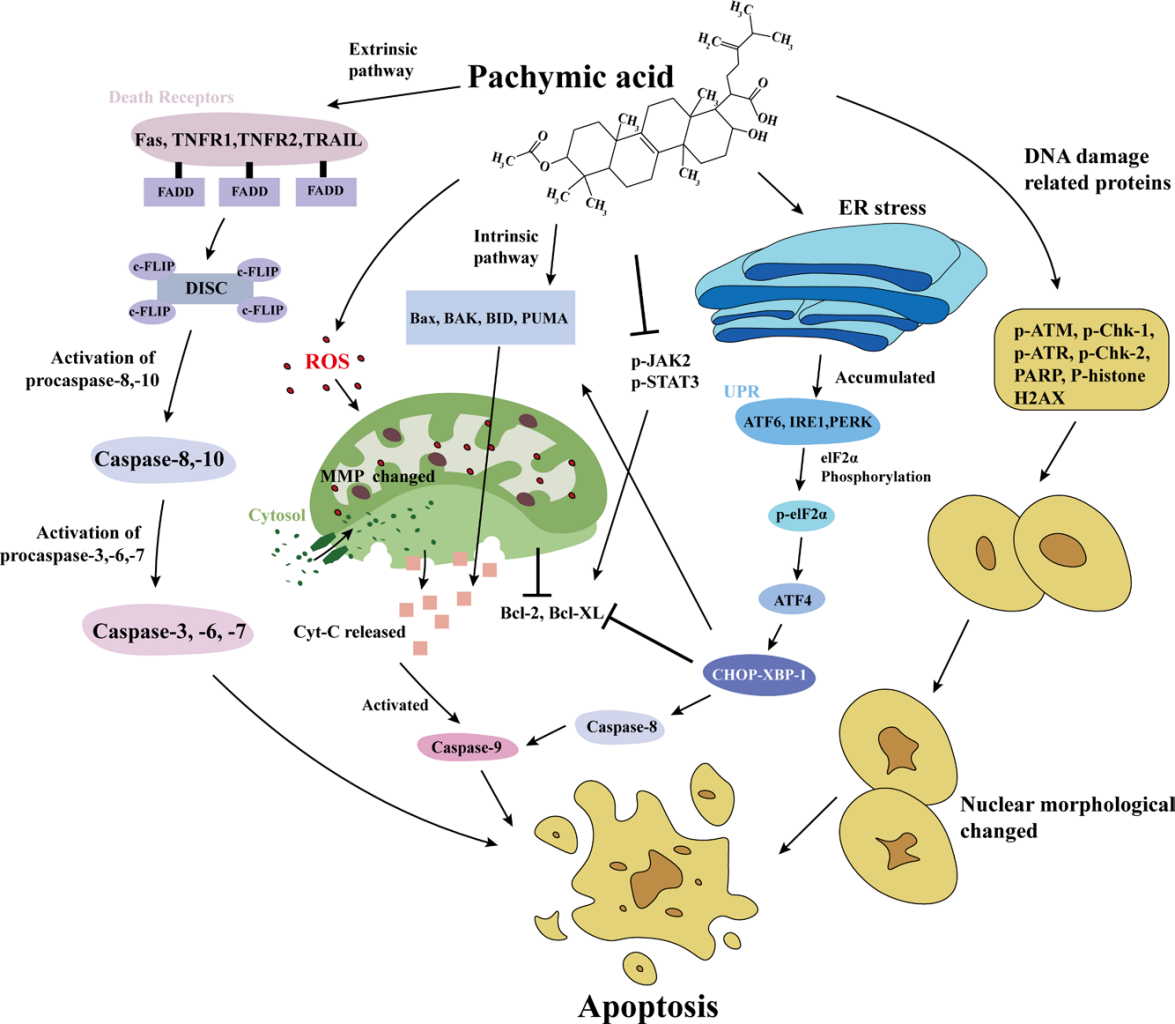
PA could induce apoptosis in cancer cells by regulating the intrinsic pathway, extrinsic pathway, ER stress, and JAK2/STAT3 pathway. PA could also promote apoptosis by altering MMP and upregulating the expression of DNA damage-related proteins. ATF = activating transcription factor, BAK = Bcl-2 antagonist killer 1, Bax = Bcl-2-associated X, Bcl-2 = B cell lymphoma-2, Bcl-XL = B-cell lymphoma-extra large, BID = BH3-interacting domain death agonist, c-FLIP = cellular FLICE (FADD-like IL-1beta-converting enzyme)-inhibitory protein, CHOP = C/EBP homologous protein, Cyt-c = cytochrome c, DICS = death-inducing signaling complex, ER = endoplasmic reticulum, Fas = factor-related apoptosis IRE1 = inositol-required protein 1, PA = pachymic acid, PARP = poly (ADP-ribose) polymerase, p-ATR = p-serine/threonine-protein kinase ATR, p-Chk = p-serine/threonine-protein kinase Chk, p-eIF2α = p-eukaryotic initiation factor 2α, PERK = protein kinase RNA like ER kinase, p-JAK = Janus kinase, p-STAT3 = p-signal transducer and activator of transcription 3, PUMA = p53-upregulated mediator of apoptosis, ROS = reactive oxygen species, TNFR = tumor necrosis factor receptor-1, TRAIL = tumor necrosis factor-related apoptosis-inducing ligand, XBP-1 = α-X-box binding protein.

The extraneous apoptosis pathway occurs as a result of the activation of proteins associated with death receptors on the cell membrane (Fas, tumor necrosis factor receptor-1, -2, and tumor necrosis factor-related apoptosis-inducing ligand ).^[37]^ Upon activation of the death-inducing signal complex downstream of the death receptor, procapsase-8 is activated.^[38]^ When the cellular FLICE (FADD-like IL-1beta-converting enzyme)-inhibitory protein is recruited at the death-inducing signal complex, caspase is triggered. In the extrinsic pathway, caspase-8, -10 activation led to caspase-3, -6, and -7 activation.^[39]^ These results indicated that PA could regulate the apoptosis of tumor cells through intrinsic and extrinsic pathways. Moreover, PA could also induce apoptosis in cancer cells in other ways (Fig. 1).

ER stress is caused by the accumulation of unfolded proteins in the ER cavity caused by ROS, hypoxia, and low nutrients in the tumor microenvironment. There are several proteins associated with ER stress, including glucose-regulated protein 78, activating transcription factors 6, 4 (ATF6, ATF4), inositol-required protein 1 (IRE1), α-X-box binding protein 1 (XBP-1), protein kinase RNA-like ER kinase (PERK), and eukaryotic initiation factor 2 (eIF2α). These proteins were overexpressed in many types of cancer cells, indicating that ER stress exists in different cancer cells.^[40–42]^ Cancer cells responded to ER stress by developing an unfolded protein response (UPR). UPR is activated by 3 ER transmembrane receptors (ATF6, IRE1, and PERK). As ER stress status worsens and UPR are unable to treat it, the accumulation of ATF6, IRE1, and PERK would cause eIF2α phosphorylated.^[43]^ P-eIF2α activated ATF4, which targeted the apoptosis effector CHOP (C/EBP homologous protein)/XBP-1.^[44]^ CHOP/XBP-1 could reduce the expression of Bcl-2 and Bcl-XL proteins and increase the expression of BAK, Bax, BIM, and PUMA.^[45]^ CHOP/XBP-1 could also activate caspase-8, thus inducing downstream caspase-9 activation.^[46]^ In a study conducted by Cheng et al^[30]^ PA was shown to induce apoptosis in pancreatic cancer cells by promoting ER stress and improving XBP-1s, ATF4, CHOP, and p-eIF2α. It was found that PA treatment increased the expression of ATF4, ATF6, XBP-1, and CHOP in lung cancer cells, promoted ER stress, and induced apoptosis.^[32]^

The Janus kinase 2/signal transducer and activator of transcription 3 (JAK2/STAT3) pathway had been confirmed to regulate cell processes in a variety of cancer cells.^[47–49]^ As the cancer microenvironment accumulates cytokines IL-6 and IL-10, STAT3 is activated by the continuous activation of cytokine receptors (vascular endothelial growth factor receptor/epidermal growth factor receptor) or nonreceptor tyrosine kinases (JAKs, Src, and Abl). STAT3 activation promoted cancer cells to encode Bcl-2, inhibited the expression of Bax/BAK, and increased the expression of cell cycle and angiogenesis factor regulator genes.^[50,51]^ Sun and Xia^[29]^ found that PA inhibited JAK2/STAT3 signaling, reduced Bcl-2 expression, and promoted apoptosis of cancer cells.

Excessive ROS production can damage mitochondria and alter mitochondrial membrane potential (MMP).^[52]^ As a result of MMP collapse, cytosol enters the mitochondrial surface osmotic transition pore, causing the mitochondrial matrix to expand and the outer membrane to rupture, which results in a large amount of Cyt-c being released into the cytoplasm, resulting in apoptosis.^[53]^ Yang et al research showed that PA promoted the accumulation of ROS in mitochondria, altered MMP, and reduced the production of adenosine triphosphate. After MMP changed, a large amount of Cyt-c was released, which induced apoptosis of cervical cancer cells.^[19]^

Studies have shown that upregulating the expression of DNA damage-related proteins, such as serine-protein kinase ATM, p-serine/threonine-protein kinase Chk (p-Chk-1 and p-Chk-2), p-serine/threonine-protein kinase ATR, poly (ADP-ribose) polymerase, and p-histone H2AX, may induce apoptosis.^[54–56]^ Bai et al^[56]^ demonstrated that PA could cause morphological changes in the nucleus, increase the phosphorylation level of proteins (p-ATM, p-Chk-1, p-serine/threonine-protein kinase ATR, p-Chk-2, poly (ADP-ribose) polymerase, and P-histone H2AX), and induce apoptosis in nasopharyngeal carcinoma cells. PA regulates DNA damage-related proteins, but little research has been conducted on its exact mechanism, and further study will be necessary to prove this hypothesis.

## 3. Cell-cycle modulation of PA

**Figure 2.**
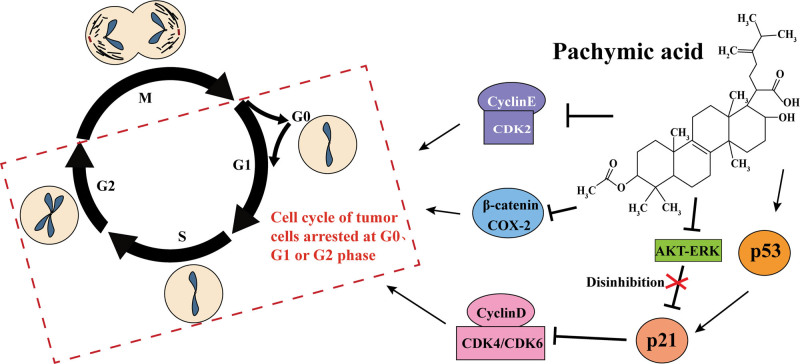
PA could cause cancer cells to enter a cell cycle arrest by inhibiting cyclin E/CDK2, COX/β-catenin. PA inhibited AKT-ERK, and the inhibition of p21 was released. PA also promoted p53; p21 was increased to degrade cyclinD-CDK4/6. AKT-ERK = protein kinase B-extracellular signal-regulated kinase, CDK = cyclin-dependent kinases, COX-2 = cyclooxygenase-2, PA = pachymic acid.

Several cell cycle mechanisms control cell division to ensure the production of 2 identical cells.^[57]^ Checkpoint control and sequential activation of cyclin-dependent kinase (CDK) regulate cell cycle progression and division.^[58]^ There are 4 phases in the cell cycle: G1, S, G2, and M. Cells in the G0 phase are also known as dormant cells, which are temporarily unable to proliferate.^[59]^ Cell-cycle progression from the G1 to the S phase may be regulated by the CyclinD-CDK4/6 complex.^[60]^ In G0/G1, p53 increases p21 levels to degrade these complexes. CyclinD, CDK4, or CDK6 gene ablation could be reduced to prevent tumor formation.^[61]^ Many studies have demonstrated that regulating the cell cycle may be a therapeutic strategy in tumors, and related drugs targeting CDK4 or CDK6 have been developed, such as Palbociclib and Ribociclib.^[62,63]^ As compared to the previous chemotherapy drugs, the side effects of these are much less severe. However, following the administration of Palbociclib, the patient developed febrile neutropenia, grade 3 stomatitis with lip swelling, periorbital edema, transaminase inflammation, and pain at the injection site.^[64]^ Taking Ribociclib may also result in neutropenia, mucositis, fatigue, gastrointestinal side effects, and even hepatobiliary toxicity.^[65]^ Studies have shown that PA inhibited cell cycle in most cases (Fig. 2; Table 2). Moreover, PA exhibited no toxic or side effects in experimental animals taken at various concentrations.^[36,28]^ PA increased p53 expression in breast cancer, promoted p21, inhibited the complex between cyclin D and CDK4/6 as well as the complex between cyclin E and CDK2, and made cancer cells stagnate in the G0/G1 phase.^[36]^ In studies conducted on ovarian cancer and GC, PA was found to arrest cancer cells in G0/G1 phase.^[29,67]^ Besides stopping the cell cycle of gallbladder cancer cells, PA also diminished the expression of oncoproteins proliferating cell nuclear antigen, intercellular adhesion molecule 1, and Ras homolog gene family, member A.^[66]^ It was also found that PA inhibited the signaling pathways AKT and ERK, while these pathways could inhibit the expression of p21 and promote cell proliferation.^[68,69]^ Some studies indicated that COX-2 (cyclooxygenase-2)/β-catenin pathway could promote the proliferation of cancer cells and regulate the cell cycle of cancer cells.^[70–72]^ Research by Gao et al^[67]^ showed that PA arrested cell cycle in the G1 phase by affecting COX-2/β-catenin pathway. However, Lu et al^[28]^ found that PA promoted GC cells to stagnate in the G1 or G2/M phase and restricted the cells to stay in the G0 phase. Their results also indicated that PA inhibited GC cell proliferation through cell cycle arrest. In their study, Lu et al detected the status of GC cells within the cell cycle but did not detect the expression of cell cycle-related proteins. Therefore, the mechanism of PA causing different cancer cells to stay in different cell cycle stages is still unclear. But what is certain is that PA causes cancer cells to enter a cell cycle arrest and limits their ability to divide.

**Table 2:** Cell-cycle modulation of PA and its derivatives.

Cell line		Study type	Mechanism	Reference
Human gallbladder cancer cells (GBC-SD)		In vitro	Cell cycle arrest at G0 phase was induced by PA. PA inhibited AKT and ERK signaling pathways.	^[66]^
Human breast cancer cells (MDA-MB-231)		In vivo and in vitro	The expression of p53 and p21 was upregulated, while cell cycle-associated cyclin D1, cyclin E, CDK2, and CDK4 were downregulated following the PA treatment. Cell cycle arrested at G0/G1 phase.	^[36]^
Human gastric cancer cells	SGC-7901 and MKN-49P	In vivo and in vitro	PA could potently inhibit GC cell growth and colony formation. PA significantly induced G1, G2/M, and inhibited G0 phase arrest.	^[28]^
	SGC-7901 cells	In vitro	PA was able to significantly inhibit the viability and induce G0/G1 cell cycle arrest.	^[29]^
Human ovarian carcinoma cells (HO-8910)		In vitro	PA caused cell cycle arrest at the G1 phase and downregulated β-catenin and COX-2.	^[67]^

AKT = protein kinase B, CDK = cyclin-dependent kinases, COX-2 = cyclooxygenase-2, PA = pachymic acid, ERK = extracellular signal-regulated kinase.

## 4. Therapeutic effect of PA combined with standard treatments of anticancer

Conventional drugs and radiotherapy are the standard treatments for cancer. It is common for clinical cancer treatments to fail due to low response rates, nonresponses, or drug resistance. Because of this, cancer is generally treated with a combination of multiple drugs. Studies combined PA with other anticancer drugs to avoid adverse reactions or toxicity caused by the combination of medications and to observe if there was multi-drug resistance (MDR). Zhang et al^[73]^ studied the therapeutic effect of PA combined with bavachin on rats with lung cancer. The results showed that PA enhanced the metabolic stability of bavachin and inhibited the transport of bavachin in lung cancer cells. P-glycoprotein (P-gp) expression in lung cancer cells was also significantly reduced by PA. A cytochrome P450 enzyme activity plays an important role in the pharmacokinetic interaction between different drugs.^[74]^ PA inhibited CYP2C9 activity in rat liver microsomes in this experiment. A previous study showed that PA is a competitive inhibitor of CYP2C9, which is responsible for the metabolism of bavachin.^[75]^ Li et al^[76]^ used P. cocos extracts (PT: PA and dehydrotumulosic acid) and doxorubicin together to treat breast cancer. In their results, the expressions of P-gp and caveolin-1 were decreased, and MDR was reversed after PT-mediated. Doxorubicin anticancer effects were amplified when combined with PT. In the above-mentioned experiments, PA was shown to be able to greatly enhance the sensitivity of cancer cells and reduce MDR by regulating P-gp.

New blood vessels are formed during tumor growth, which provides nutrients for cancer cells.^[77]^ Thus, inhibiting malignant tumor growth can be achieved by weakening angiogenesis and endothelial remodeling and regression. A combination of multi-walled nanotubes (MWNTs) and PA was applied to chicken embryos by Ma et al.^[78]^ It was also found that PA/MWNTs had a strong inhibitory effect on angiogenic activity and reduced the expression of matrix metalloproteinase-3 in chorioallantoic membrane tissues. Matrix metalloproteinase-3 is a major protease associated with tumor metastasis.^[79]^ This experiment demonstrated that MWNTs decreased vascular branching, while PA inhibited vascular growth. It is, therefore, possible to inhibit angiogenesis and prevent tumor invasion through the combination of these 2 approaches.

As GC is not detected until advanced stages, many patients need to undergo surgical resection.^[80]^ It has been demonstrated in clinical studies that patients undergoing surgery along with adjuvant chemotherapy and radiotherapy had a significantly longer overall survival rate.^[81]^ However, their prognosis was still poor. The combination of PA with radiotherapy has been shown to have a therapeutic effect on GC.^[20]^ GC cells were sensitive to radiation both in vitro and in vivo when PA was administered. Bax upregulation was mediated by hypoxia-inducible factor-1alpha inhibition in PA-treated cells. They demonstrated that PA-induced radiation sensitivity of GC cells was mediated by hypoxia-inducible factor-1alph inhibition. This study suggested more possibilities for PA in cancer treatment.

## 5. Conclusion

Cancer incidence rates have increased worldwide in recent years, and cancer treatment methods have also improved. According to existing research, PA extracted from P. cocos is an anticancer agent. The anticancer action of PA was achieved through a variety of mechanisms. PA caused cancer cells to undergo apoptosis. PA stopped the cell cycle of cancer cells, limiting their division. PA is combined with anticancer drugs and radiotherapy to increase the sensitivity of cancer cells. PA could also be combined with biomaterials to weaken the formation of blood vessels in cancer tissue. The safety of PA has also been confirmed in vivo, as no toxic or adverse reactions were observed. As there are few studies on the effect of PA on cancer, the current number of references is limited, and all of them show the positive effect of PA on cancer treatment. Consequently, PA has the potential to be a valuable source of effective anticancer drugs. In the future, more clinical trials and patent research will be needed to prove its effectiveness.

## Author contributions

**Conceptualization:** Yubo Xiao.

**Funding acquisition:** Yubo Xiao, Ming Li.

**Investigation:** Yubo Xiao.

**Writing – original draft:** Yubo Xiao, Zhaotun Hu.

**Writing – review & editing:** Yubo Xiao, Zhaotun Hu.

**Data curation:** Hang Liu, Xinglin Jiang.

**Formal analysis:** Hang Liu, Taimei Zhou.

**Validation:** Xinglin Jiang.

**Project administration:** Haiying Wang.

**Methodology:** Heng Long.

**Resources:** Heng Long.

**Software:** Ming Li.

**Visualization:** Ming Li.
